# Multisite Evaluation of Point of Care CD4 Testing in Papua New Guinea

**DOI:** 10.1371/journal.pone.0112173

**Published:** 2014-11-26

**Authors:** Malin Malagun, Gideon Nano, Caroline Chevallier, Ragagalo Opina, Gola Sawiya, Joseph Kivavia, Albina Kalinoe, Kathalina Nathaniel, Oscillah Kaminiel, John Millan, Andrea Carmone, Mary Dini, Theresa Palou, Kum Topma, Evelyn Lavu, Jessica Markby

**Affiliations:** 1 Clinton Health Access Initiative, Port Moresby, Papua New Guinea; 2 National Department of Health, HIV Division, Port Moresby, Papua New Guinea; 3 Heduru Clinic, Port Moresby General Hospital, Papua New Guinea; 4 Central Public Health Laboratory, HIV Section, Port Moresby, Papua New Guinea; 5 Pathology Department, Port Moresby General Hospital, Port Moresby, Papua New Guinea; 6 Central Public Health Laboratory, Quality Assurance Section, Port Moresby, Papua New Guinea; 7 National Department of Health, Port Moresby, Papua New Guinea; 8 Clinton Health Access Initiative, Goroka, Papua New Guinea; 9 Pathology Department, Goroka General Hospital, Papua New Guinea; 10 Asaro District Health Centre, Eastern Highlands Province, Papua New Guinea; 11 Central Public Health Laboratory, Papua New Guinea; Emory University School of Medicine, United States of America

## Abstract

Laboratory-based CD4 monitoring of HIV patients presents challenges in resource limited settings (RLS) including frequent machine breakdown, poor engineering support and limited cold chain and specimen transport logistics. This study assessed the performance of two CD4 tests designed for use in RLS; the Dynal assay and the Alere PIMA test (PIMA). Accuracy of Dynal and PIMA using venous blood was assessed in a centralised laboratory by comparison to BD FACSCount (BD FACS). Dynal had a mean bias of −50.35 cells/µl (r^2^ = 0.973, p<0.0001, n = 101) and PIMA −22.43 cells/µl (r^2^ = 0.964, p<0.0001, n = 139) compared to BD FACS. Similar results were observed for PIMA operated by clinicians in one urban (n = 117) and two rural clinics (n = 98). Using internal control beads, PIMA precision was 10.34% CV (low bead mean 214.24 cells/µl) and 8.29% (high bead mean 920.73 cells/µl) and similar %CV results were observed external quality assurance (EQA) and replicate patient samples. Dynal did not perform using EQA and no internal controls are supplied by the manufacturer, however duplicate testing of samples resulted in r^2^ = 0.961, p<0.0001, mean bias = −1.44 cells/µl. Using the cut-off of 350 cells/µl compared to BD FACS, PIMA had a sensitivity of 88.85% and specificity of 98.71% and Dynal 88.61% and 100%. A total of 0.44% (2/452) of patient samples were misclassified as “no treat” and 7.30% (33/452) “treat” using PIMA whereas with Dynal 8.91% (9/101) as “treat” and 0% as “no treat”. In our setting PIMA was found to be accurate, precise and user-friendly in both laboratory and clinic settings. Dynal performed well in initial centralized laboratory evaluation, however lacks requisite quality control measures, and was technically more difficult to use, making it less suitable for use at lower tiered laboratories.

## Introduction

In 2004, PNG became the fourth country in the Asia Pacific region to declare a generalised HIV epidemic, with the highest prevalence of HIV (1.3% in 2007) in the Oceania region [1]. Although data recently reported by the PNG National Department of Health (NDOH) in 2010 indicate a modest decline in the prevalence rate (0.92%), the number of new infections continues to increase, particularly in rural and remote areas where services are weakest [2]. The roll out of ART needs to be synchronized with increased access to CD4 cell count testing. Currently in PNG, it is estimated that less than 30% of HIV positive individuals who need regular testing have access to centralized CD4 [3].

CD4 cell count testing is a major determinant of ART eligibility among adults and is recommended by the World Health Organization (WHO) for monitoring all HIV positive patients globally, when viral load testing is unavailable and for assessing co-infections [4]. The need for expensive and sophisticated instruments, highly trained staff and fresh whole blood can limit access to CD4 testing beyond centralised laboratory services. Long turn-around time for results from centralised CD4 testing can result in a significant loss to follow-up of patients, especially in settings where patients travel long distances to access health services [5], [6], [7], [8].

In PNG, flow cytometric CD4 testing (BD FACS) has been significantly hampered by widespread problems including regular optics fluidics malfunctions linked to power failures, poor sample quality, poor laboratory climate control and incomplete maintenance. In addition, cold chain logistics to transport reagents and samples from rural settings to provincial laboratories is challenging. Collectively this has resulted in reduced test result quality, poor access and significant loss to follow up of patients.

In the last few years, the market landscape for CD4 testing has been rapidly changing to address many of these challenges commonly experienced in RLS. Alternative, low-cost tests that circumvent the need for expensive, sophisticated equipment have emerged for use both in lower tier laboratories and at the point of care [9]. One such assay, the Dynabeads T4 Quant Kit (Dynal), (Life Technologies, Melbourne, Australia) assay requires only a microscope making it suitable for lower tiered laboratories. The Dynal assay has been validated in field settings in India, West Africa and Fiji and has been used for clinical monitoring in countries including Kenya, Indonesia, India and Fiji [10], [11].

Newer approaches to CD4 testing however have focussed on developing point of care (POC) CD4 tests that can be used in remote, rural clinical settings where the majority of HIV positive individuals reside. It has been demonstrated that availability of a CD4 test result at the same time as HIV testing significantly increases ART initiation rates [12], [13], [14]. Although several POC CD4 platforms, all requiring some form of instrument, have emerged on the market, there is currently only one WHO prequalified CD4 test available: the PIMA (Alere, Brisbane, Australia) [15]. PIMA does not require cold chain logistics, manual sample processing, or maintenance and can be performed by an operator with limited technical skills. The cost of the portable device will vary but is approximately USD 8000, and runs for up to eight hours from a rechargeable battery. PIMA provides a rapid result turn-around time (less than 20 minutes), however only one patient sample can be processed at a time. There have been a number of evaluation studies of the PIMA test in both developed laboratory and field settings using blood obtained by venepuncture or finger prick showing excellent correlation with flow cytometry results [16], [17], [18], [19], [20], [21], [22], [23], [24], [25].

The purpose of this study was to carry out pre-implementation field evaluations of low cost CD4 technologies. We selected a robust low-tiered laboratory test, the Dynal assay and a POC test, the PIMA. We assessed the performance both assays (accuracy and precision) using venous blood in field laboratories compared to flow cytometry. We also graded the tests according to operational characteristics (cost, ease of use, result turn-around time and performance in external quality assurance). In addition, we assessed the performance of the PIMA in remote clinical settings.

## Methodology

### Study participants and blood specimens

HIV positive, adult participants were recruited from one urban (Heduru HIV clinic at Port Moresby General Hospital, Port Moresby, n = 139) and two rural clinics (Asaro District Health Centre and Kainantu Rural Hospital both in the rural PNG n = 98). In order to ensure a range of CD4 count results, equal numbers of HIV positive patients on ART and HIV positive patients immediately prior to commencement of ART were enrolled simultaneously (CD4 count range of study participants: 25–1157 cells/µl). Participants provided written informed consent for collection of venous blood for analysis of CD4. Inclusion criteria included written clinic records of an HIV positive diagnosis and age >18 years. TB or other co-infection information was not collected. Approximately 5 ml of venous blood was collected from each patient into K_3_-EDTA vacutainer tubes; samples were tested for CD4 count within six hours of collection. This study was approved by the Papua New Guinean Research Advisory Committee and the Medical Research Advisory Committee (RAC Approval Number RES08 0015 and MRAC Approval Number 10.28).

### CD4 enumeration

Five PIMA were used in the study, located at one of the above clinics (n = 3) as well as one at the urban reference laboratory (Central Public Health Laboratory, Port Moresby), and one at a rural laboratory (Goroka Pathology Laboratory, Goroka General Hospital). At each site laboratory or clinical staff were trained for one day in the operation of the PIMA according to manufacturer instructions and provided with training aides and training in clinical workflow for point of care testing. A designated study laboratory technician was present during the duration of the study period at each testing site to supervise specimen testing, data entry and quality assurance.

For the PIMA CD4 testing, 25 µl samples of fresh whole venous blood was loaded onto the PIMA reagent cartridges using EDTA treated capillary tubes. The PIMA cartridge was capped immediately and loaded into the PIMA within 5 minutes for processing. Precision of test results was measured by performing the PIMA test in duplicate/replicate on patient samples.

In order to assess whether there was a difference between the accuracy of testing performed by laboratory technicians and clinicians, the same samples were tested using PIMA operated by laboratory technicians and clinicians in both urban and rural settings to compare the similarity of results obtained.

To assess the accuracy of PIMA results in comparison with the predicate method, the same blood samples were also tested in the laboratory using BD FACS. Here, 50 µl of fresh whole venous blood was added to the BD FACS CD4/CD3 reagent tube and the fix/no lyse protocol was followed according to manufacturer's instructions.

Separate samples (each 125 µl) were tested in duplicate using the Dynal Quant T4 assay (Dynal, Compaiegne, France) according to the manufacturer's instructions as well as by BD FACSCount system (BD Biosciences, Brisbane, Australia) to assess accuracy.

### Quality control

Internal control beads (low and normal) supplied by the manufacturers (Alere and BD FACS) were run daily for their respective assays at each testing facility (laboratories and clinics) prior to testing of patient specimens. No internal controls were available for the Dynal assay. Control bead data were stored on the hard drive of each machine and analysed for performance throughout the duration of the study to determine the mean, standard deviation and percentage coefficient of variation (%CV). An external quality assurance (EQA) panel of two stabilized whole blood specimens provided by the Public Health Agency of Canada, Quality Assurance Scheme for Immunology (QASI) was assessed using each of the three CD4 tests [26]. Each EQA specimen was tested repetitively (n = 19 and 20) to determine the mean, standard deviation and percentage coefficient of variation for each EQA specimen. EQA specimens were also compared to the global consensus value provided by the QASI organiser.

### Statistical Analysis

All statistical analysis was carried out using Prism software, version 5.0d. Accuracy, bias and limits of agreement (LOA) of the technique was assessed by comparing the absolute CD4 count values obtained using the PIMA and Dynal compared to the predicate method (BD FACS) using Bland Altman [27] and linear regression analyses was used to calculate the correlation coefficient (r^2^). Mann-Whitney tests were used to test for significant differences between the two techniques. Precision of results using the PIMA was assessed by calculating the percentage coefficient of variation (%CV) and mean difference.

## Results

### Quality Assurance and precision of the PIMA

The performance of PIMA using manufacture-provided internal control beads when performed by either laboratory or clinical staff at each of the five sites was assessed with resulting a mean coefficient of variation of 10.34% (low beads mean = 214.24 cells/µl and 8.29% (normal beads mean = 920.73 cells/µl (Table 1A). Precision was also assessed by testing replicates (n>19) EQA panels samples (provided by QASI) resulting in similar coefficient of variations (intra-operator variability) 12.24%, sample mean = 165.63 cells/µl and 7.59%, sample mean 383.21 cells/µl Table 1B). Reproducibility of PIMA was also assessed by running a proportion of the specimens in duplicate (n = 29) with resulting coefficient of variation (intra-operator variability) = 5.25%, sample mean = 272.75 cells/µl compared to the predicate method (n = 20) which resulted in a coefficient of variation (intra-operator variability) = 7.29%, sample mean 275.90 cells/µl (Table 1C).

**Table 1:** Quality Assurance and precision of PIMA.

A.	Low Beads	Normal Beads	B.	Sample 1	Sample 2
			n	19	20
**Site 1 Urban Laboratory**			**Mean ± SD**	165.63±20.28	383.21±29.09
**n**	20	19	**% CV**	12.24	7.59
**Mean ± SD**	218.10±15.42	852.32±59.49	**BD FACS (n = 2)**	171	381
**% CV**	15.42	6.98	**QASI**	153	374
**Site 1 Urban Clinic**					
**n**	17	21	**C.**	**BD FACS**	**PIMA**
**Mean ± SD**	222±19.51	898.76±89.22	**n**	20	29
**% CV**	8.79	9.93	**Mean ± SD**	272.75±14.14	275.90±17.04
**Site 3 Rural Laboratory**			**% CV**	5.25	7.29
**n**	15	15	***P*** ** value**	0.957	0.882
**Mean ± SD**	216.6±23.47	955.07±85.47			
**% CV**	10.83	8.95			
**Site 5 Rural Clinic 1**					
**n**	11	13			
**Mean ± SD**	226.5±12.92	993.58±83.24			
**% CV**	5.7	8.38			
**Site 5 Rural Clinic 2**					
**n**	10	10			
**Mean ± SD**	188±20.62	903.90±65.30			
**% CV**	10.97	7.22			

A. Internal Quality control using manufacturer provided bead standard controls Normal (mean 957, range 670–1244 cells/µl) and Low (mean 192, range 132–252 cells/µl). (Site 1: Urban Laboratory, Site 2: Urban Clinic, Site 3: Rural Laboratory, Site 4: Rural Clinic, Site 5: Rural Clinic). B. QASI External Quality Assurance Panel each sample tested in replicate by a single laboratory technician at the reference laboratory, Central Public Health Laboratory (CPHL). C. Assessment of assay precision using replicates from the same blood sample run in duplicate by a single laboratory technician. n = sample size,

B. SD = standard deviation, CV = coefficient of variation, QASI = Quality Assurance Scheme for Immunology (Public Health Agency of Canada).

### Urban and rural performance of the PIMA compared to the BD FACS

**Figure 1:**
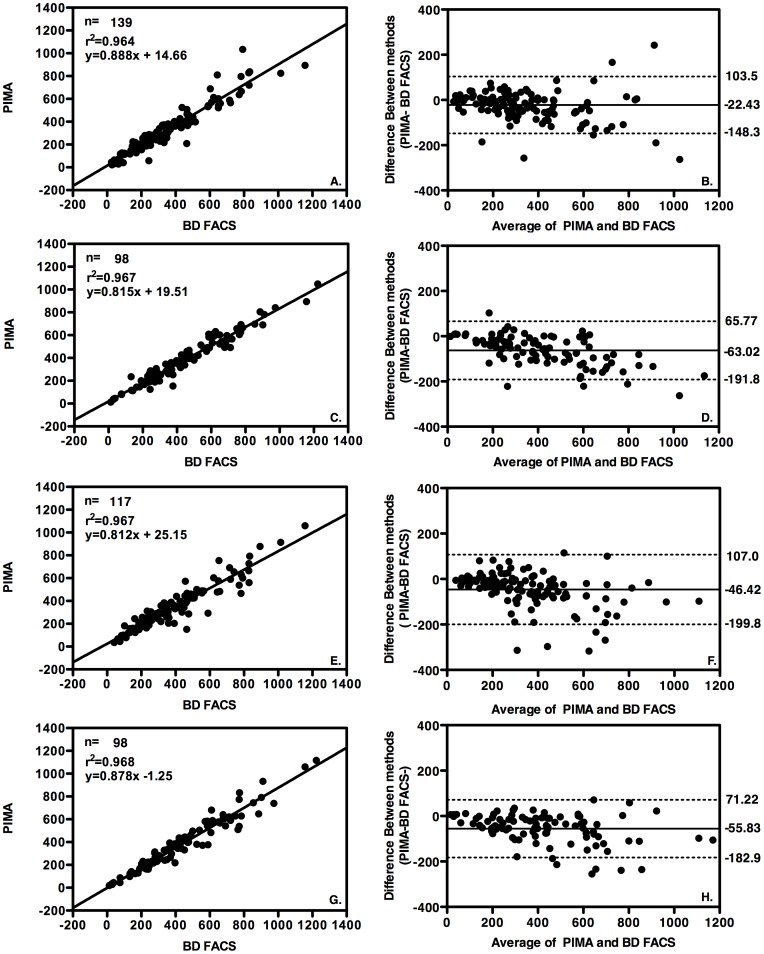
Accuracy of PIMA compared to BD FACS. Linear regression analysis plots (right column) and Bland Altman bias plots (left column) with upper and lower 95% limits of agreement (LOA) shown as broken lines and mean bias shown as an unbroken line. A. and B. PIMA versus BD FACS both performed at an urban (reference) laboratory, by a single laboratory technician, C. and D. BD FACS versus PIMA both performed at the rural laboratory by a single laboratory technician, E. and F. BD FACS performed by laboratory technician at an urban laboratory PIMA versus PIMA performed by clinical staff at an urban clinic, G. and PIMA. BD FACS performed at by laboratory technician at a rural laboratory versus PIMA performed at two rural clinics by clinical staff.

We assessed the accuracy of the PIMA using venous blood on five devices; one located in an urban laboratory, one in an urban clinic, one in a rural laboratory and two in rural clinics. In both laboratories, each blood specimen was also tested using BD FACS. The PIMA results in both urban and rural laboratory settings run by a single laboratory technician at each site were compared to the predicate method in each case using linear regression analysis (CPHL n = 139, mean = 330.60 cells/µl, r^2^ = 0.964, p<0.0001 and GGH n = 98, mean = 383.24 cells/µl, r^2^ = 0.967, p<0.0001) Figure 1 (A & C) and Bland-Altman analysis indicated a mean bias of −22.43 cells/µl and −63.02 cells/µl in these respective laboratory settings (Figure 1 B & D).

We also assessed the accuracy of PIMA devices when operated at urban and rural clinics by trained clinicians and compared the results to the predicate method performed at the referral laboratory. Linear regression analysis (Figure 1 E & G) resulted in a coefficient of determination (r^2^) of 0.967, p<0.0001, (n = 117, mean CD4 count = 323.65 cells/µl) at the urban clinic and r^2^ = 0.968, p<0.0001 (n = 98, mean CD4 count 390.44 cells/µl) at the two rural clinics. Bland-Altman bias analysis (Figure 1 F & H) indicated a mean bias of −46.42 and −55.83 cells/µl in these respective clinic settings. Using the cut-off of 350 cells/µl compared to BD FACS, PIMA had an overall sensitivity of 88.85% and specificity of 98.72% (Table 2A). The error rate for PIMA was recorded at the urban clinic and observed to be 5.13% (n = 6/117) and was comprised of operator/machine based errors described in more detail in Table S1. Using the CD4 cut off of 350 cells/µl [4], a total of 0.44% (2/452) of patient samples were misclassified as “no treat” (358 and 372 cells/µl respectively) and 7.30% (33/452) misclassified as “treat” using PIMA compared to the predicate method (Table 2A).

**Table 2:** Clinical Misclassification Analysis using PIMA and Dynal compared to BD FACS.

A.					B.			
**1. Urban Laboratory**		**PIMA**				**BD FACS**	**PIMA**	**Delta**
		**<350**	**>350**	**Total**	**1. Urban Lab**	**323**	**370**	**47**
**BD FACS**	**<350**	85	1	86		355	305	−50
	**>350**	3	50	53		369	333	−36
	**Total**	88	51	139		433	347	−86
	**Sensitivity**	96.59	98.04	**Specificity**	**2. Urban Clinic**	**333**	**383**	**50**
						413	342	−71
**2. Urban Clinic**		**PIMA**				359	205	−154
		**<350**	**>350**	**Total**		398	342	−56
**BD FACS**	**<350**	58	1	59		392	203	−189
	**>350**	14	44	58		356	303	−53
	**Total**	72	45	117		355	274	−81
	**Sensitivity**	80.56	97.78	**Specificity**		361	253	−108
						477	283	−194
**3. Rural Laboratory**		**PIMA**				438	302	−136
		**<350**	**>350**	**Total**		590	293	−297
**BD FACS**	**<350**	60	0	60		397	331	−66
	**>350**	8	30	38		449	342	−107
	**Total**	68	30	98		465	151	−314
	**Sensitivity**	88.24	100.00	**Specificity**		433	344	−89
					**3. Rural Lab**	433	347	−86
**4. Rural Clinics**		**PIMA**				413	343	−70
		**<350**	**>350**	**Total**		412	340	−72
**BD FACS**	**<350**	60	0	60		354	324	−30
	**>350**	8	30	38		376	154	−222
	**Total**	68	30	98		410	343	−67
	**Sensitivity**	88.24	100.00	**Specificity**		421	315	−106
						369	297	−72
**Total All PIMA**		**PIMA**			**4. Rural Clinics**	433	344	−89
		**<350**	**>350**	**Total**		413	344	−69
**BD FACS**	**<350**	263	2	265		452	331	−121
	**>350**	33	154	187		376	297	−79
	**Total**	296	156	452		354	338	−16
	**Sensitivity**	88.85	98.72	**Specificity**		397	218	−179
						369	264	−105
						350	246	−104
**Dynal Urban Laboratory**		**Dynal**				**BD FACS**	**Dynal**	**Delta**
		**<350**	**>350**	**Total**	**Dynal Urban Lab**	405	312	−93
**BD FACS**	**<350**	70	0	70		494	315	−179
	**>350**	9	22	31		526	328	−198
	**Total**	79	22	101		377	231	−146
	**Sensitivity**	88.61	100	**Specificity**		382	305	−78
						405	305	−100
						501	316	−185
						359	284	−75
						397	301	−96

A. 2×2 Tables CD4 count compared to BD FACS clinical misclassification using 350 cells/µl cut off for determination of ART Eligibility at each site and total for PIMA 1,2,3,4 and Dynal 5. B. BD FACS and PIMA CD4 count results for misclassified samples from each site 1,2,3,4 and 5. Dynal. Values in bold font indicate samples misclassified by PIMA as non-treat (<350 cells/µl) and values in normal font indicate those samples misclassified by PIMA or Dynal as treat (<350 cells/µl).

### Comparison of laboratory and clinical operator performance using the PIMA

**Figure 2:**
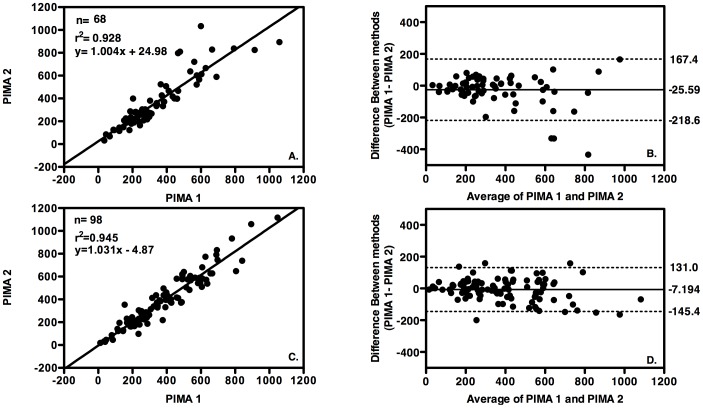
Operator Precision of PIMA testing. Linear regression analysis plots (right column) and Bland Altman bias plots (left column) with upper and lower 95% limits of agreement (LOA) shown as broken lines and mean bias shown as an unbroken line. A and B PIMA 1 operated by laboratory technicians at an urban (reference) laboratory, versus PIMA 2 operated by clinicians at an urban clinic, C and D PIMA 1 operated by laboratory technicians a rural laboratory compared to PIMA 2 operated by clinicians at 2 rural clinics.

We also compared the results obtained by PIMA operators with different levels of professional training (laboratory technicians versus clinician in both urban and rural centres i.e. inter-operator variability) and performed analysis using linear regression and Bland-Altman bias analysis. In the urban settings, the coefficient of determination (r^2^) was 0.928, p<0.0001 (n = 68) with a mean bias of −25.59 cells/µl towards laboratory testing (Figure 2 A & B). The same analyses of results obtained in the rural laboratory and rural clinics resulted in a coefficient of determination (r^2^) of 0.945, p<0.0001 (n = 98) and mean bias towards laboratory testing of −7.194 cells/µl) (Figure 2 C & D).

### Performance of the Dynal assay compared to the BD FACS

**Figure 3:**
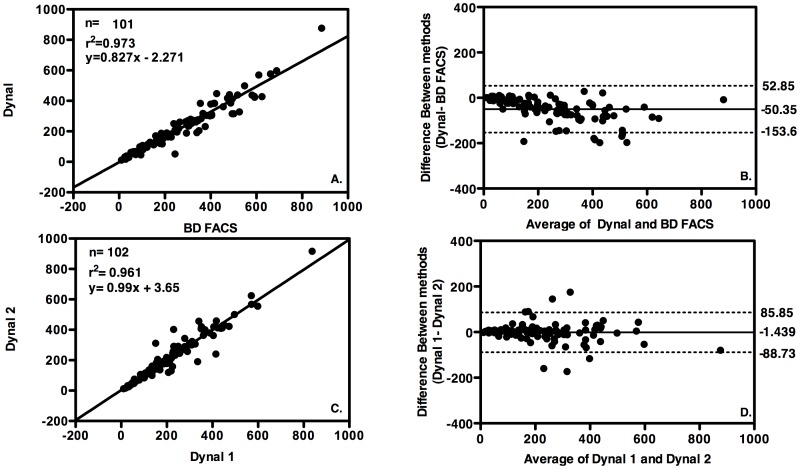
Accuracy and Precision of Dynal assay. Linear regression analysis plots (right column) and Bland Altman bias plots (left column) with upper and lower 95% limits of agreement (LOA) shown as broken lines and mean bias shown as an unbroken line. A. and B. Dynal accuracy versus BD FACS in urban (reference) laboratory C. and D. Precision of measurements using the Dynal assay performed in duplicate by the same laboratory technician in urban (reference) laboratory.

The performance of the Dynal assay was assessed for accuracy at the urban reference laboratory compared to the predicate method (BD FACS) using linear regression and Bland Altman analysis. The mean CD4 count for the samples tested was 227.36 cells/µl, n = 101 and the coefficient of determination (r^2^) was 0.973, p<0.0001 with a mean bias = −50.35 cells/µl towards Dynal. (Figure 3 A & B). Precision was not assessed using the Dynal assay using EQA samples or internal controls as EQA panels would not be successfully testing using the Dynal assay and no internal controls are provided by the manufacturer. Reproducibility was measured by running all samples twice with the same operator and resulted in a coefficient of repeatability of r^2^ = 0.961, p<0.0001, a mean bias = −1.44 cells/µl. A total of 8.91% (9/101) were misclassified as “treat” and no patient samples were misclassified as “no treat” using the Dynal assay compared to the predicate method (Table 2A). Using the cut-off of 350 cells/µl compared to BD FACS, Dynal had a sensitivity of 88.61% and a specificity of 100%.

### Operational Characteristics

In our urban setting we assessed several operational characteristics of the assays including cost, throughput, time for processing, result turn-around time, technical difficulty, operator skill, equipment, reagents, sample and cold chain requirements, technical problems, electricity requirement, suitability with EQAS, internal control provisions. Although PIMA was more expensive than Dynal for initial equipment costs, running costs were comparable between PIMA and Dynal (USD 8 and 10 respectively). Dynal required additional reagents and buffers to be made up, whereas no additional buffers or reagents were required for PIMA. The average throughput per day was lower for Dynal than PIMA in our setting (mean 5 and 15 in an 8 hour day respectively). Eye fatigue associated with long microscope time and long preparation time (23 steps) were the rate-limiting steps associated with performing Dynal, whereas PIMA required only 4 steps with minimal complexity for processing. The turn-around time for results to get to patients was significantly shorter for PIMA than Dynal due to the fact that results were available within 20 minutes at the clinic for PIMA, whereas samples had to be referred to the laboratory for processing using Dynal and patients had to return to the clinic for results. Dynal was found not to be suitable for EQAS in our setting and the kit does not contain internal controls whereas PIMA performed well with EQAS and the kit included reagent internal controls as well as separate bead controls.

## Discussion

This study was the first of its kind in the Asia Pacific region to evaluate point of care CD4 technologies in rural and remote field laboratory and clinic settings. In our setting, we were able to reproduce the accuracy and precision of PIMA CD4 testing previously reported only in controlled, developed laboratory settings in this region [18]. Staff from clinics have a basic level of clinical training operation with some recent experience using the rapid point of care tests for HIV diagnosis, however regardless of the operator skill level, we observed excellent correlation at all sites evaluated.

Evidence from evaluation and implementation of PIMA in Africa indicated a relatively high error rate (>10% in South Africa [21] and 8.1% in Uganda [17]). It is possible that we may have seen a greater errors (5.12% in our setting) and misclassification if we had used finger prick sampling rather than venous blood. Glencross et al, 2012 observed unacceptable precision using capillary blood collection (mean SIM CV 28.4% compared to predicate testing [21]. Mwau et al and Diaw et al also reported decreased sensitivity and specificity of PIMA when capillary blood was used compared to venous blood [19], [20]. Therefore in this study and in the implementation of PIMA in PNG, venous blood was used as the specimen of choice to ensure quality of test results and to build on existing strong venous blood specimen collection practices in country. The demand for using finger prick is growing in PNG in light of the roll out of POC rapid testing for HIV diagnosis, which also uses finger prick sampling. Others have reported the coupling of HIV rapid testing with POC CD4 testing can lead to improved accuracy, likely due to the fact that health care workers in these settings are highly skilled and experienced in collecting finger prick specimens [24]. Therefore it will be important to evaluate the accuracy of finger prick sampling in PNG to assess its applicability in our setting.

Since this evaluation of point of care CD4 testing in PNG, PIMA was prequalified by the WHO in 2010 [15] prompting its approval for use in PNG by the National Department of Health, who, together with partners, have begun implementation of PIMA at 33 sites nationally. Many lessons can be shared from the implementation, including the adoption of key site selection criteria assessments to ensure optimal operation of PIMA in the national testing program. Assessments should include availability of reliable electricity, provision of ART on-site (ideally) or by referral systems, adequate staff volume and capacity, lack of or inadequate access to CD4 testing (geographically or long turn-around times), and sites that require rapid CD4 results such as antenatal clinics or VCT sites.

Centrally coordinated standardised clinician training, certification, supervisory site visits, and data collection are critical components in the successful implementation of POC CD4 testing in such a setting. In addition, as with all POC testing, the need for quality management [6], including participation in EQAS [28] and data management, cannot be overstated. Although comparatively inexpensive, any POC test using equipment is subject to breakdown, highlighting the importance for technical engineering support for PIMA implementation in rural settings or for the implementation of tests that require no instrumentation.

We observed that the Dynal assay performed well in a centralised laboratory in the PNG context. However, the assay requires a relatively high level of skill for operation including competency in microscopy, reliable refrigeration and freezers are required for reagent storage and a functional microscope and assay specific equipment are required. The Dynal assay is relatively time consuming compared to other CD4 assays due to the manual preparation and cell counting required and therefore is not suited to a high throughput laboratory setting with an upper limit in our clinic/lab setting of five patients per day. In addition, Dynal was found to be unsuitable for use with the External Quality Assurance Scheme (EQAS) specimens from the Canadian Quality Assurance Scheme for Immunology (QASI), due to the fact that QASI samples are whole blood that is stabilized during preparation, the binding site of cell surface proteins targeted by anti-CD4 monoclonal antibodies used in the Dynal kit is altered yielding no result. This makes the use of the Dynal assay in routine diagnostics less ideal. Neither Dynal nor PIMA delivers results in terms of percentage, making neither test ideal for the care of HIV infected children under five. The new BD FACS software however allows both absolute count and CD4 percent which also sets a new benchmark which it is hoped point of care technologies will follow. The PIMA test however is low cost and requires only low technology equipment and may therefore be useful in low throughput, low resource research laboratories as others have indicated [11], [19].

Whilst still currently dominated by laboratory-based testing, the current market for CD4 monitoring is shifting towards increased POC testing. The wider use of POC tests is likely to have a dramatic effect on HIV clinical management in RLS and particularly for those living in remote rural areas where access to laboratory testing is non-existent or limited. Studies in Africa have demonstrated that POC CD4 testing reduces loss to follow up, cuts time to initiation of ART and increases the rate of ART initiation from 33% to 64% [13]. The findings of this and similar studies can inform programs in countries in other RLS and help provide access to appropriate and accurate CD4 testing.

## Supporting Information

Table S1 **Comparison of Operational Characteristics CD4 assays assessed in this study.** Characteristics associated with operation of the three CD4 assays were assessed during the study period at the urban laboratory (BD FACS and Dynal) and urban clinic (Pima). Costs (USD-United States dollars) are approximate based on local costing for supply in Papua New Guinea at the time of the study and may vary according to volume and country of supply. The throughput per day and results turn-around time was based on data collected during the study period assessed retrospectively and reflect the normal urban clinic and laboratory work flow according to the number of staff available to process samples in this setting and existing results reporting mechanisms and time frames. The errors observed using Pima included the following error codes observed in the urban clinic during n = 117 tests; Invalid test error 850 (n = 1), Gaiting error 940 (n = 2), Channel filling error 810 (n = 2), Volume error, 201 (n = 1) These results are representative of error rates and types observed at all sites where Pima was used in this study. EQAS = External Quality Assurance Scheme, assessed the use of EQAS panels supplied by QASI (Quality Assurance Scheme for Immunology) EQAS program provided free of charge by the Canadian Public Health Agency.
(DOCX)

## References

[1] PNG National Department of Health (2007) The 2007 STI, HIV and AIDS Annual Surveillance Report.

[2] PNG National Department of Health (2010) The 2010 STI, HIV and AIDS Annual Surveillance Report.

[3] Chevallier C (2012) Personal Communication ed.

[4] World Health Organization (2013) Consolidated guidelines on the use of antiretroviral drugs for treating and preventing HIV infection: Recommendations for a public health approach. World Health Organization Geneva. 24716260

[5] Peeling RW, Mabey D (2010) Point-of-care tests for diagnosing infections in the developing world. Clinical microbiology and infection: the official publication of the European Society of Clinical Microbiology and Infectious Diseases 16:1062–1069. 10.1111/j.1469-0691.2010.03279.x 20670288

[6] Peter T, Badrichani A, Wu E, Freeman R, Ncube B, et al (2008) Challenges in implementing CD4 testing in resource-limited settings. Cytometry Part B, Clinical cytometry 74 Suppl 1:S123–130. 10.1002/cyto.b.20416 18348208

[7] Zachariah R, Reid SD, Chaillet P, Massaquoi M, Schouten EJ, et al (2011) Viewpoint: Why do we need a point-of-care CD4 test for low-income countries? Tropical medicine & international health: TM & IH 16:37–41. 21371207 10.1111/j.1365-3156.2010.02669.x

[8] Marinucci F, Medina-Moreno S, Paterniti AD, Wattleworth M, Redfield RR (2011) Decentralization of CD4 testing in resource-limited settings: 7 years of experience in six African countries. Cytometry Part A: the journal of the International Society for Analytical Cytology 79:368–374. 21495181 10.1002/cyto.a.21064

[9] Larson B, Schnippel K, Ndibongo B, Long L, Fox MP, et al (2012) How to estimate the cost of point-of-care CD4 testing in program settings: an example using the Alere PIMA Analyzer in South Africa. PloS one 7:e35444. 22532854 10.1371/journal.pone.0035444 PMC3331987

[10] Truett AA, Letizia A, Malyangu E, Sinyangwe F, Morales BN, et al (2006) Efficacy of Cyto-Chex blood preservative for delayed manual CD4 testing using Dynal T4 Quant CD4 test among HIV-infected persons in Zambia. Journal of acquired immune deficiency syndromes 41:168–174. 16394848 10.1097/01.qai.0000191282.77954.fc

[11] Plate MM, Louzao R, Steele PM, Greengrass V, Morris LM, et al (2009) Evaluation of the blood stabilizers TransFix and Cyto-Chex BCT for low-cost CD4 T-cell methodologies. Viral immunology 22:329–332. 19811090 10.1089/vim.2009.0027

[12] Faal M, Naidoo N, Glencross DK, Venter WD, Osih R (2011) Providing immediate CD4 count results at HIV testing improves ART initiation. Journal of acquired immune deficiency syndromes 58:e54–59. 21857356 10.1097/QAI.0b013e3182303921

[13] Jani IV, Sitoe NE, Alfai ER, Chongo PL, Quevedo JI, et al (2011) Effect of point-of-care CD4 cell count tests on retention of patients and rates of antiretroviral therapy initiation in primary health clinics: an observational cohort study. Lancet 378:1572–1579. 21951656 10.1016/S0140-6736(11)61052-0

[14] Larson BA, Schnippel K, Ndibongo B, Xulu T, Brennan A, et al (2012) Rapid point-of-care CD4 testing at mobile HIV testing sites to increase linkage to care: an evaluation of a pilot program in South Africa. Journal of acquired immune deficiency syndromes 61:e13–17. 22659650 10.1097/QAI.0b013e31825eec60 PMC3458178

[15] World Health Organization (2011) WHO Prequalification of Diagnostics Programme PUBLIC REPORT, Product: PIMA CD4 Test, Number: PQDx 0099-032-00. World Health Organization Geneva.

[16] Westerman L (2009) Evaluation of PIMA CD4 Assay. Oral Presentation presented at the International AIDS Symposium Cape Town, South Africa.

[17] Manabe YC, Wang Y, Elbireer A, Auerbach B, Castelnuovo B (2012) Evaluation of portable point-of-care CD4 counter with high sensitivity for detecting patients eligible for antiretroviral therapy. PlOS one 7:e34319. 22536323 10.1371/journal.pone.0034319 PMC3334961

[18] Sukapirom K, Onlamoon N, Thepthai C, Polsrila K, Tassaneetrithep B, et al (2011) Performance evaluation of the Alere PIMA CD4 test for monitoring HIV-infected individuals in resource-constrained settings. Journal of acquired immune deficiency syndromes 58:141–147. 21709568 10.1097/QAI.0b013e31822866a2

[19] Diaw PA, Daneau G, Coly AA, Ndiaye BP, Wade D, et al (2011) Multisite evaluation of a point-of-care instrument for CD4(+) T-cell enumeration using venous and finger-prick blood: the PIMA CD4. Journal of acquired immune deficiency syndromes 58:e103–111. 21909029 10.1097/QAI.0b013e318235b378

[20] Mwau M, Adungo F, Kadima S, Njagi E, Kirwaye C, et al (2013) Evaluation of PIMA Point of Care Technology for CD4 T Cell Enumeration in Kenya. PLoS ONE 8:e67612 doi:67610.61371/journal.pone.0067612 23825674 10.1371/journal.pone.0067612 PMC3692483

[21] Glencross D, Coetzee L, Lwrie D (2010) Performance evaluation of PIMA CD4 point-of-care analyzer, Johannesburg, South Africa. XVII International AIDS Conference Vienna, Austria.

[22] Glencross DK, Coetzee LM, Faal M, Masango M, Stevens WS, et al (2012) Performance evaluation of the PIMA point-of-care CD4 analyser using capillary blood sampling in field tests in South Africa. Journal of the International AIDS Society 15:3. 22284546 10.1186/1758-2652-15-3 PMC3310849

[23] Herbert S, Edwards S, Carrick G, Copas A, Sandford C, et al (2012) Evaluation of PIMA point-of-care CD4 testing in a large UK HIV service. Sexually transmitted infections Part 88. Sexually Transmitted Infections 6:413–417. 10.1136/sextrans-2012-050507 22544309

[24] Mtapuri-Zinyowera S, Chideme M, Mangwanya D, Mugurungi O, Gudukeya S, et al (2010) Evaluation of the PIMA point-of-care CD4 analyzer in VCT clinics in Zimbabwe. Journal of acquired immune deficiency syndromes 55:1–7. 20622679 10.1097/QAI.0b013e3181e93071

[25] Thakar M, Mahajan B, Shaikh N, Bagwan S, Sane S, et al (2012) Utility of the point of care CD4 analyzer, PIMA, to enumerate CD4 counts in the field settings in India. AIDS research and therapy 9. 10.1186/1742-6405-9-26 PMC3503578 22998738

[26] Bergeron M, Ding T, Houle G, Ares L, Chabot C, et al (2010) QASI, an international quality management system for CD4 T-cell enumeration focused to make a global difference. Cytometry Part B, Clinical cytometry 78:41–48. 10.1002/cyto.b.20487 19598239

[27] Bland JM, Altman DG (1986) Statistical emthods for assessing agreement between two methods of clinical measurement. Lancet 1:307–310. 2868172

[28] Baum LL, Crowe S, Landay AL (2007) Advances in CD4 cell enumeration in resource-poor countries. Current opinion in HIV and AIDS 2:234–240. 19372892 10.1097/COH.0b013e3280ef6909

