# Supplementary material for: Multisite Evaluation of Point of Care CD4 Testing in Papua New Guinea
Source: PLoS One. 2014 Nov 26;9(11):e112173. doi: 10.1371/journal.pone.0112173 (PMC4245096; doi:10.1371/journal.pone.0112173)
Supplement: Table S1 — Comparison of Operational Characteristics CD4 assays assessed in this study. Characteristics associated with operation of the three CD4 assays were assessed during the study period at the urban laboratory (BD FACS and Dynal) and urban clinic (Pima). Costs (USD-United States dollars) are approximate based on local costing for supply in Papua New Guinea at the time of the study and may vary according to volume and country of supply. The throughput per day and results turn-around time was based on data collected during the study period assessed retrospectively and reflect the normal urban clinic and laboratory work flow according to the number of staff available to process samples in this setting and existing results reporting mechanisms and time frames. The errors observed using Pima included the following error codes observed in the urban clinic during n = 117 tests; Invalid test error 850 (n = 1), Gaiting error 940 (n = 2), Channel filling error 810 (n = 2), Volume error, 201 (n = 1) These results are representative of error rates and types observed at all sites where Pima was used in this study. EQAS = External Quality Assurance Scheme, assessed the use of EQAS panels supplied by QASI (Quality Assurance Scheme for Immunology) EQAS program provided free of charge by the Canadian Public Health Agency. (DOCX) [file pone.0112173.s001.docx]

| **Characteristic** | **BD FACS** | **PIMA** | **Dynal** |
| --- | --- | --- | --- |
| **Approx. Cost per test USD** | 8 | 9 | 10 |
| **Approx. Set up cost USD** | 30,000 | 8000 | 1500 |
| **Throughput/day (mean)** | 20 | 10 | 5 |
| **Time for processing** | 2 hours | 20 mins | 1 hour |
| **Result turn-around time** | 7 days | 20 mins | 7 days |
|  |  |  |  |
| **Technical difficulty (number of steps) + <4 ++ 5-8 +++>9** | +++ (17) | +(4) | +++ (23) |
| **Operator skill level** | Laboratory technician | Laboratory technician/Clinician | Laboratory technician |
| **Equipment required** | BD FACS Machine, vortex, automated pipette | Device only, comes with printer | Light microscope, magnet MPC T4 Quant, tube rotating wheel, haemocytometer, automated pipettes |
| **Sample required** | EDTA Venous blood within 24 hours | EDTA Venous within 6 hours | EDTA Venous blood within 24 hours |
| **Additional Reagents required** | BD FACS Saline solution BD FACS Control beads  FACSClean and FACSRinse | PIMA Standard Control beads (low and normal) | Acridine Orange Stain, Sternheimer, Malbin Stain, Turck Stain, Washing Buffer |
| **Cold Chain required for reagents** | Yes | No | Yes |
| **Technical Problems** | Two sheath blockages (cleared on same day) and one laser failure, stopped the study for 6 weeks | 4.6 % error rate | Blurry eyes due to long count times= limit number of tests/day to 5 |
|  |  |  |  |
| **Electricity requirement** | Mains | Mains/Rechargeable Battery | Mains |
| **Suitable for use with EQAS** | Yes | Yes | No |
| **Internal controls provided by manufacturer** | Yes | Yes | No |
